# Shellfishing on foot and the road to defeminization in Galicia (Spain)

**DOI:** 10.1007/s40152-021-00228-z

**Published:** 2021-07-15

**Authors:** María de los Ángeles Piñeiro-Antelo, Xosé M Santos

**Affiliations:** Department of Geography, University of Santiago de Compostela, Santiago de Compostela, Spain

**Keywords:** Empowerment, Fishing sector, Galicia, Professionalization, Shellfish harvesting on foot, Women

## Abstract

Fishing resources, as well as fishing activities and policies, are in a state of permanent change, therefore transforming the living and working conditions of coastal and fishing populations. The gender perspective is relevant to understand the challenges faced by men and women in the fishing sector. Galicia (Spain) is one of the main fishing regions in the EU and with the largest number of women working in the fishing sector, especially in shellfishing on foot. Shellfishing on foot, an artisanal and traditional activity for the cultivation and extraction of mainly bivalve molluscs, represents 7% of gross value added (GVA) and 17% of the employment of the Galician fishing sector as a whole. Since the 1960s, a process of regulation and modernization of shellfishing on foot—more than 95% of which is carried out by women—has led to a sharp decrease in the number of shellfish gatherers. The regulatory processes and the professionalization of the sector have resulted in a strong decline in female employment, but, at the same time, women feel empowered and regard their jobs as dignified work. Our objective—through the analysis of the local permits granted to carry out this activity—focuses on the study of the consolidation of this process and has tried to highlight the ways in which patriarchy perpetuates the hegemonic position of men evidenced, for example, by a progressive masculinization of this activity with increasing economic profitability and social prestige.

## Introduction

Fisheries and fisheries-related activities still play a crucial role in the economy and employment of some European countries and regions. This is the case in Spain, which accounts for 25% of fishing employment in Europe and which, together with Portugal, Italy, and Greece, represents almost three quarters of the European Community’s fishing employment (European Commission 2018).

The strong masculinization of fishing activities in the world (European Commission 2002) and the invisibility and lack of weight of women in decision-making organizations (Alonso-Población and Siar 2018) are also well known. As an example of this in different territories, and motivations, Frangoudes and Gerrard (2019) cite studies in Norway, Malawi, and Peru, which highlight the norms, cultural traditions, or symbolic barriers to explain the obstacles that women face when accessing certain fishing activities (Kleiber and Harris 2015; Manyungwa-Pasani and Hara, 2019; Delaney et al. 2019).

In Spain, as in most other countries, fishing is heavily masculinized; however, there are a higher number of women working in the sector (European Commission 2002, 2018). Galicia, in the North West of the Iberian Peninsula, is the Spanish region with the highest concentration of female employment in the fishing sector, at approximately 67% of Spain’s total (European Commission 2019a, b). Even so, in Galicia fishing, extractive activities are heavily masculinized (Frangoudes 2013). The latest official surveys reveal that there are 470 women working in extractive fishing in Galicia, 1118 in marine aquaculture, and 2633 in shellfish harvesting on foot, which represent 4.13%, 21.38%, and 69.7%, respectively, of the total employment in Galicia in each subsector of the fishing economy (Ocupesca 2017; IGE 2019)

The literature about gender in fisheries tries to provide more insight about women’s tasks and practices, their roles, their relations to men, their situations in different places or contexts, and some of the changes they have faced. The goal of this knowledge, as stated by Frangoudes, Gerrard, and Kleiber is that equal rights between women and men within fisheries are also a part of sustainable fisheries (Frangoudes et al. 2019, p. 120).

Fishing resources and the activity linked to them, as well as the sectoral policies, are in permanent transformation generating changing living and working conditions for coastal populations dependent on fishing (Frangoudes and Gerrard 2019). Therefore, studying labour relations in depth from a gender perspective is important to understand not only the common challenges but also, and especially, the specific challenges faced by men and women in the fishing sector.

In the case of Galicia, there is a large amount of literature on the importance of women’s work in fishing and their traditional leading role in activities related to shellfishing or the canning industry (Marugán Pintos 2004, 2010, 2012, Martínez 2016, Broullón Acuña 2011). Our starting point is that advances in the professionalization of shellfish activity on foot, that is, in the “process of turning shellfishing on foot into an activity that is the main source of income for the people who do it, and that allows them to receive appropriate social benefits” (Xunta de Galicia 1992, 46). This objective has resulted in the transformation of the activity from the seasonal and occasional harvesting of resources to cultivation with different operations distributed throughout the year and strongly regulated (Santasmarinas 2010).

The professionalization of shellfish farming and the economic crisis of 2008 have been two of the causes that have caused a trend towards masculinization (Martínez 2019, Red Española de Mujeres en el Sector Pesquero (REMSP) 2016). Martínez García (2016, p. 3) points out that some forms of discrimination against women have diminished “thanks to the professionalisation processes in which these workers are involved in and regularizing their trades to achieve improvements in their working conditions as well as increasing their quality of life. (…) However, other inequalities are still strongly rooted, revealing that the steps taken are insufficient and that they require not only their effort, but also the commitment of the institutions and the population”.

In this context, this paper, based on research at a local level, tries to follow the process of the professionalization of the shellfishing on foot sector, historically very feminized and socially disregarded. While progress is being made in that direction, and as a consequence of this process, we are witnessing the consolidation of masculinization in this activity. The research also highlights the non-reciprocity of the phenomenon, emphasizing the non-existence of an equivalent trend of women accessing highly masculinized activities such as extractive fishing.

This work collects academic literature focused on the efforts to dignify this profession, with specific contributions highlighting the perpetuation of the patriarchy, since the long process of economic and social “dignification” of the profession runs in parallel with the expulsion of the most fragile women (older and those with more family responsibilities) followed by the masculinization process that we are currently witnessing.

With this focus in mind, a methodological section is included, and then, we will review the literature on gender divisions in the labour markets with a special focus on the sea. A case study is focused on the field of shellfish gathering on foot in Galicia, Spain. Here, we will analyse the importance of the fishing economy to the region and the changes experienced in shellfish gathering on foot, while trying to understand the causes that have generated those changes.

## Sources and methodology

**Fig. 1:**
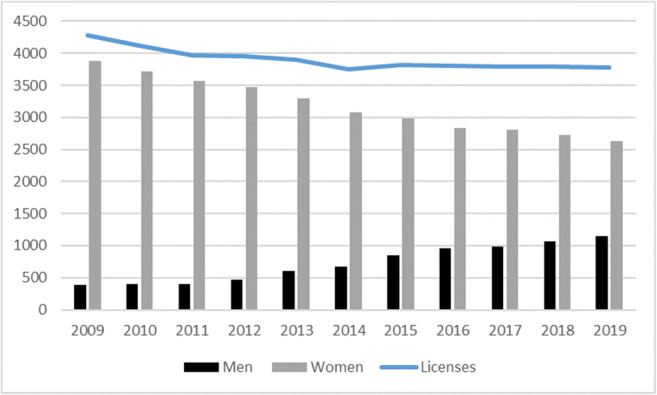
Permits for shellfish on foot in Galicia (2009–2019). Source: Galician Institute of Statistics. www.ige.eu

**Fig. 2:**
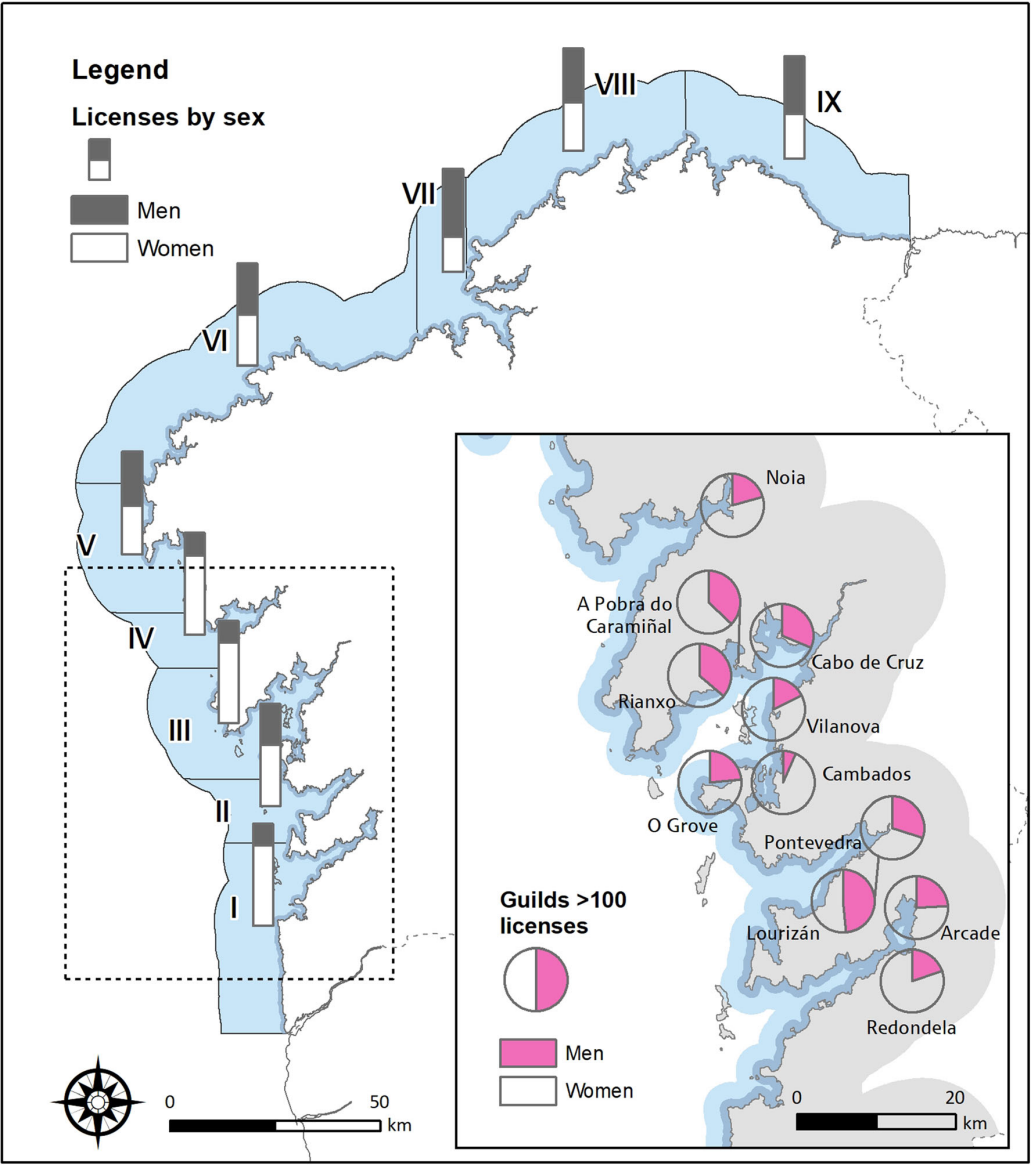
Permits for shellfish on foot by zones and guilds (2019). Source: Galician Institute of Statistics. www.ige.eu

This paper has analysed the technical documentation related to the legal framework of on-foot shellfish gathering in Galicia, to know the evolution of the laws and norms that regulate the activity. This analysis has been completed using a statistical analysis of data published by the Galician Ministry of the Sea, regarding the evolution of permits for shellfishing on foot granted since 2009, the year in which the series of data disaggregated by sex begins (Fig. 1). These data are available at a regional level (Galicia), and since 2011, also at lower scales: (i) by nine Galician production zones (Fig. 2) and (ii) at the local level by Fishermen’s Association (guilds), the bodies responsible for the resource management plans of shellfish resources. The guilds are sectoral, non-profit public sector corporations that represent the interests of professionals in the fishing sector. These Galician guilds collaborate with regional governments in matters of the management of the fishing sector, with a commitment to contribute to local development, social cohesion, and sustainability (Law 9/1993, of 8 July, of the Fishermen’s Guilds of Galicia; Law 3/2001, of 26 March, of Maritime Fishing of the State, Spain).

The level of disaggregation of the data presents a series of limitations when it comes to deepening the analysis of the process of the incorporation of men into shellfishing on foot, because data disaggregated by sex is not available for each typology, that is determined by the target species being harvested (e.g. general, barnacle, and polychaete).

In addition, a detailed analysis of the literature on gender and labour segmentation in fisheries has been carried out for this study. This includes, first, a review of articles that analyse the role of women in the fishing economy and their importance in maintaining traditional activities linked to the sea. Secondly, theories related to the division of labour by sex are considered, alongside those that try to explain the causes and implications of gender-related changes in the labour market.

Finally, a review of the local press that specialize in maritime affairs in Galicia has been carried out; this is a valuable source of information for data on retirements and new entries in shellfish gathering permits at a local level, details that in many cases are not published in official statistics.

## Gender and labour segmentation in fisheries

### The gender social systems under which women participate in fishing and fisheries

A large body of research has developed over recent decades highlighting gender segmentation in the labour market (Blau and Wallace 1979; Walby 1988; Bradley 1993; Hanson and Pratt 1995; McDowell 2009). Other studies have also been concerned with occupational segregation in fishing communities (Yodanis 2000; Hapke 2001; Porter and Mbezi 2010; Kleiber and Harris 2015), particularly with regard to South-East Asia and some regions of Africa where fisheries have great economic and social weight. Academic articles, books, official reports, and scientific meetings—from the First Global Workshop on Women in Aquaculture organized by the Food and Agriculture Organization (FAO) in Rome (1987) to more recent ones—have multiplied, and highlight, among other issues, the important role of women in relation to fishing activities and the lack of information and visibility of their function (Sunderarajan 2002; Williams et al. 2002; García Negro and Zotes Tarrío 2006; Porter and Mbezi 2010; Dowling 2011; Kleiber and Harris 2015).

Women’s participation in fisheries-related activities is often linked to economic precariousness (Kleiber and Harris 2015). Poverty is a feature of many coastal communities (Macfadyen and Corcoran 2002; Hapke 2001; Porter and Mbezi 2010), and so women’s work is a key contribution to household income. Following the argument of Sunderarajan (2002), it is necessary to pay more attention to the change, from a focus on women in fisheries to gender and fisheries (Williams et al. 2006; Williams 2008). The latter implies not only evidencing gender roles but also understanding the subordinate position that women occupy in terms of the performance of their activities. Authors such as Lambeth et al. (2002) highlight the need to work with the entire community involved at every stage of the productive chain; separating men’s and women’s tasks prevents us from seeing the problem as a whole, just as it does if we project an exclusively sectoral vision. Broullón Acuña (2011) analyses the subsidiary character given to shellfish in Galicia and frames it in terms of sexual dimorphism between land and sea, in which the latter is associated with harshness, heroism, and virility and is therefore masculine (Martínez García 2017). This sea/man vs. land/woman dichotomy reflects a discriminatory structure in gender relations (Broullón Acuña 2011).

Kleiber and Harris (2015), in their gender study of small-scale fisheries (including non-boat fisheries such as the gleaning of invertebrates in intertidal spaces), review the content of published research. In addition to evidencing and trying to understand the causes of the important gaps in data provision, especially quantitative data, they defend and demonstrate the relevance of gender analysis in small-scale fisheries “not only for socio-economic concerns, but also to gain a more comprehensive and robust understanding of the human role in marine ecosystems” (Kleiber and Harris 2015: 558). While recognizing that gender roles are dynamic and open to change, some of the aspects addressed by Kleiber and Harris (2015) in their bibliographical review raise the point that women tend to work in intertidal spaces where the capture of invertebrates predominates, as in the case of bivalves in Galicia; such spaces also include the practice of other post-harvest activities (Sunderarajan 2002).

In order to explain why women work in these near-home habitats, Kleiber and Harris (2015) compile, from the existing literature, multiple examples that allow us to have a clear idea of the situation. Thus, authors like Tekanene (2006) point out that the activities that women tend to carry out are characterized by their location close to the home, being part-time and requiring low costs in terms of investment and maintenance. In short, their aim is to combine professional and domestic tasks, including the possibility of taking their children with them while they work on the sandbanks. Women’s fishing and fisheries labour are often viewed as simply part of their domestic tasks, the catch was destined for family food, and the surplus was sold or exchanged. In addition, this work is associated with craft characteristics that require little expenditure on work equipment. It is clear, therefore, that this division of roles reflects the classic patterns of discrimination in which the role of women as housewives and life support for the family is given priority over their role as fish workers. This is compounded by other prejudices such as the social rejection of women’s inclusion on board ships, a workplace that is almost exclusively reserved for men; there can be no doubt that this is the case in Galicia. In fact, on a scale ranging from large vessels that fish in international waters to small vessels that fish close to the coast, the presence of women is almost irrelevant in the former and very insignificant in the latter (García Negro and Zotes Tarrío 2006, Ocupesca 2017).

### Changes in this gender social systems and the cost of these changes to the women and men in fisheries

Another characteristic of women’s work in sea-related activities is their dedication to tasks and to the harvesting of species that are less lucrative and generate less economic benefit (Porter and Mbezi 2010). In the case of Tanzania, Porter and Mbezi (2010) point out that as the value of a catch increases, the field is increasingly monopolized by men, leading to the deprivation and marginalization of women.

The masculinization of jobs that were considered female has not received the same attention as the feminization of typically male jobs (Lindsay 2007). Bradley (1993) describes three stages in gender changes in the labour market: infiltration (occurs when only a few men are present in a woman’s occupation), invasion (occurs when many men enter an occupation that women have not completely abandoned), and takeover (occurs when an occupation is redefined as “men’s work”). Meanwhile, Lindsay (2007) identifies four key issues for understanding the process: scarcity of alternative employment, opportunities for faster promotion, changes in working conditions, and the incorporation of technology. In both cases, it is important to point out that we are not only facing a process of substitution; the implications are greater, assuming, for example, an increase in the status of that work so that it is considered “real” work (Lindsay 2007).

In short, if the incorporation of women into previously masculinized jobs does not take place under equal conditions, as evidenced by many official reports (European Commission 2019b; International Labour Organization 2018), the masculinization of what are considered women’s jobs also reveals structural issues in which power takes a leading role. Walby (1988) warns of the relationship between policies designed and implemented by administrations and the sexual segregation of the labour market. In this sense, Lindsay (2007) points out that historical evidence suggests that the most powerful professions exclude and delegitimize other professions.

Women’s fisheries labour in Galicia is concentrated in the most precarious and worst paid activities such as the processing and selling of fishery and aquaculture products (Gago and  Ardora Formación 2004). Additionally, there is strong gender segregation in the fish and aquaculture processing industry, where women have the lowest paid and least stable jobs (Red Española de Mujeres en el Sector Pesquero (REMSP) 2018). In the case of shellfishing on foot, as soon as the conditions in which the activity takes place improve, it is regarded as professional work with higher monetary returns, which therefore improve its social recognition and trigger the masculinization process (Martínez 2019, Red Española de Mujeres en el Sector Pesquero (REMSP) 2016).

## The importance of fishing and shellfishing in Galicia

### Galicia’s shellfishery evolution

Galicia is one of the most important fishing regions in the European Union, with the highest employment level and economic dependence on fishing (Salz and Macfadyen 2007; Surís-Regueiro and Santiago 2014). Within the sector, the activity with the greatest relative importance in terms of income and employment is sea fishing, which accounts for 73% of gross value added at basic prices (GVA bp) and almost 60% of full-time equivalent employment (FTE), compared to aquaculture, which represents almost 20% of GVA bp and 23% of employment. Finally, shellfish farming on foot, the traditional subsector for women’s employment in coastal communities, accounts for 7% of GVA bp and 17% of FTE (Surís-Regueiro and Santiago 2014). There are large differences between Spanish coastal regions in terms of the importance of women in fishing, with Galicia being the region where the percentage of women is highest, 31.5% of those in Galicia associated with Special Regime for the Sea of the Social Security (SRS).

The importance of women’s work at sea has always been underestimated, largely due to the serious shortcomings of fisheries statistics, which stem from the informal nature of a significant part of the activities carried out in coastal communities. Therefore, for decades, women were absent from statistical counts (Aldrey and Lois 2001; Negro et al. 2006). Their work in the fishing sector has been very important for the maintenance of family economies, although in most cases it was carried out outside the formal economy. In this sense, Barnes and Christophers (2018) discuss the socially constructed concept of economy and the power of statistics to legitimize a country’s wealth. Indeed, García Negro and Zotes Tarrío highlight the poor quality of the document prepared by the European Commission entitled *The role of women in the fisheries sector* (European Commission 2002), particularly in relation to Galicia, pointing out that this document, when used as a tool for decision-making, can lead to erroneous conclusions.

Despite the absence of statistics and the invisibility of women’s economic contribution, women have been present in fishing and shellfishing activities, especially in shellfish gathering on foot, sewing fishing nets, mussel farming, algae harvesting, and in the process of the sale and distribution of fish and aquaculture products (Santos 2001). In addition, in many cases, they have been involved in the administrative and management tasks of vessels engaged in artisanal fisheries. In all of these activities, many of these women have remained in the informal sphere; consequently, their contribution has not been quantified and has been underestimated. It was not until the final decades of the twentieth century, with the professionalization of trades such as net mending or shellfish gathering on foot, and the awareness of women of their role as sea workers, that their activity has begun to be quantified in a more accurate way (Marugán 2010; Piñeiro 2013, 2015); this has allowed them to be incorporated into the so-called formal economy.

Shellfishing in its two forms, on foot and afloat, refers to the collection of shellfish (mainly bivalve molluscs) in the intertidal zone in marine waters. Such activities are undertaken under the shared management of the Galician regional government and fishermen’s associations (guilds), who carry out intense regulatory, supervisory, and control activities. Traditionally, shellfish gathering afloat is carried out by men in a small boat, whereas shellfish gathering on foot is carried out by women, generally without boats and using very rudimentary tools (Mahou 2008).

This is a traditional craft activity that is halfway between harvesting and extensive aquaculture. Most of the work is done with elementary production methods on the sandbanks of the intertidal zones and is mostly part-time employment (Martínez Ferreiro et al. 1998) (Pérez 2010). In Galicia, as in other parts of Europe, seafood harvesting has been a subsistence activity carried out for centuries by women and children on the shore, which is considered to be a public space that is open to the whole community. Demand for these products has been low, and they have been considered to have little market value, despite being an important source of food with a markedly seasonal character (Frangoudes et al. 2008).

### Overall patterns of temporal and geographic change

Since the middle of the twentieth century, national and regional administrations have made several attempts to organize the activity, with the aim of avoiding the over-exploitation of the shellfish banks. Management instruments that have been used include activity permits, time limitations on extraction (bans), and prohibitions on the harvesting of immature shellfish through the establishment of minimum sizes. The progressive tightening of regulations and permit requirements has had a direct impact on the number of workers in the sector, a figure that has fallen considerably. While in the 1974–1975 season, the number of workers was around 60,000 (González Vidal 1980), 15 years later in the 1990–1991 season, this number had fallen to 16,000 (Pardellas 1991). Since 1993, regulations have been further tightened, so that in 1996 there were 7852 mollusc gatherers on foot throughout Galicia, most of them women (Frangoudes et al. 2008). The number of workers has continued to decrease progressively as shellfish gathering on foot has been professionalized. Thus, in 2019, this activity employed 3777 people, of which 2633 were women (IGE 2019).

The social, cultural, and economic importance of shellfish in Galicia has been the focus of numerous publications and research (Meltzoff 1995; Martínez Ferreiro et al. 1998; Gago and  Ardora Formación 2004; Marugán 2004, 2010, 2012; García Negro and Zotes Tarrío 2006; Santasmarinas 2006; Frangoudes et al. 2008; Pérez 2010), in which the role of women is always highlighted.

In her doctoral thesis, Martínez (2016) applies a gender perspective to the experiences of female fishing workers in Galicia and the Basque Country. This author analyses the long and difficult process of the professionalization of shellfishing activity and the consequent female empowerment. However, despite the enormous achievements made, she also shows that female fishing workers’ empowerment “is less at the collective level, which is the level with real transformative potential to subvert the domination exercised over women” (Martínez 2016, p. 122) and that there is also a lack of ideologization, in the sense that there is no strong gender awareness, that prevents further progress in structural change to the patriarchal society.

### Drivers of change

The professionalization process of shellfishing on foot began in the last few decades of the twentieth century and was closely linked to the development of regional competences, on the one hand, and the entry of Spain into the European Economic Community and the subsequent arrival of funds associated with the Common Fisheries Policy, on the other.

As Martínez (2016) also points out, the context of democratic governance is important for understanding the changes experienced. Progress in regionalization in Spain has meant the progressive transfer of competences to the Galician government. In 1981, the first Galician Minister of Fisheries was appointed, and, since then, the regional administration has been very involved in organizing the use of fishery resources with the aim of making a more rational use of them. In 1982, the Plan for the Management of Fishing and Shellfish Resources in Galicia (PORPMG) was passed, which gave an in-depth diagnosis of the sector’s situation and proposed solutions to the problems identified (Xunta de Galicia 1992). The principles formulated in the PORPMG were included in Law 6/93 on Fisheries, which specified Galicia’s competence in the field of fisheries. These two instruments, the plan and the law, made it possible to consolidate the professionalization of the sector. Thus, between 1992 and 1993, all Galician fisheries legislation was revised in order to recast it and facilitate its application. Between 1993 and 1994, 14 decrees were passed that served to systematize all the regulations in force in the field of fishing and adapt them to the regulations of the European Community.

This regulatory management and control action by regional government led to an unprecedented change in shellfishing on foot, which until then had been characterized by poor professional and technological training, a feminized and ageing workforce, minimal division of labour, and low investment and poor commercialization (Mahou 2008). Another serious problem affecting shellfishing on foot is poaching, which is widespread and is sometimes accepted by the community in situations of poverty or social exclusion (Ballesteros and Rodríguez-Rodríguez 2018).

The regional management of funds associated with the Common Fisheries Policy allowed for a large part of them to be invested in the management of the Galician fishing sector, and therefore in the professionalization of women shellfish workers, mainly through training courses and the introduction of cultivation techniques for a more rational exploitation of resources.

The professionalization of the sector affected women in particular as they were the majority of workers shellfishing on foot. The men, traditionally known as shellfishermen, are afloat in boats dedicated to artisan fishing and with alternating gear throughout the year, developing their activity with a fully professional character. The process of the professionalization of shellfish harvesting on foot was based on the introduction of mollusc farming techniques, the development of new production technologies, the adaptation of work to market demands, and the improvement of shellfishers’ productivity (Mahou 2008, p. 81). Galicia’s sectoral policy aimed to turn shellfishing on foot into a source of income and stable employment for the women involved in this activity. Thus, strict criteria were established to obtain operating permits (training, working days and minimum sales, an obligation to contribute to social security and pay taxes, among other things), and the system of temporary closures was replaced by a system of alternating different tasks that allowed the activity to be carried out throughout the year in cultivation areas (Frangoudes et al. 2008; Pérez 2010). Thus, shellfish gatherers on foot had first the possibility, and then, in 1993, the obligation, of making social insurance contributions as part of the Special Regime for the Sea (SRS), with increasing numbers making contributions. According to official data from the Galician government, in 2000, 90% of the workers in the subsector were contributing, and today all workers pay social insurance contributions.

Together with regulatory activity in relation to shellfish gathering on foot, the Galician administration favoured the organization and empowerment of women through the “Shellfishwomen on foot Meetings”. Since the first meeting in 1995, annual meetings have been held to promote the process of professional change and personal transformation of the women workers, with the aim of making them aware of the need for training to improve their working conditions and their capacity to become agents of social transformation. The regional government organized 124 professionalization courses between 1996 and 1997, which were attended by 1747 women shellfish harvesters, promoting the conversion of shellfish harvesters into growers and professionals (Meltzoff 1995; Marugán Pintos 2012).

At the beginning of this professionalization process, shellfishing on foot was, in most cases, complementary to the main activities of the family unit and a way of obtaining an occasional income, often through the exchange of goods in kind. A large part of the catches were not destined to be sold but to be consumed by the family. Currently, the activity is in the hands of professionals, organized in associations that, together with the guilds, and under the supervision of the Galician Administration, plan and control the activity. The empowerment of women has been an essential element in this process, which has also contributed to improving the social value of the activity (Frangoudes et al. 2008).

## Results

### Loss of some shellfisherwomen on foot

This process has meant that a large number of women who had been carrying out this activity in a traditional way, and who were not prepared to face the challenge of professionalization, abandoned shellfishing on foot. Most of the older women, those with less training, or those with greater family burdens, were not able to cope with becoming professionals and were deprived of the possibility of continuing with the activity. This circumstance did not pose a problem of particular concern to the regional administration, since the excessive number of shellfish gatherers was considered to be an obstacle to the modernization of the activity (Xunta de Galicia 1992). Therefore, no measures were ever taken to alleviate the socio-economic problems caused by this, nor were the repercussions for the maritime cultural heritage considered (Piñeiro 2015).

But, unlike what happens in the whole of Spain, where the number of women affiliated to the Social Security Sea Regime has increased progressively, in the case of Galicia, there is no increment in the labour activity of women in the fishing sector, since they accounted for 26% of affiliates in 2009 and 24% in 2019. Even between 2009 and 2019, the number of affiliations decreased by 11.8% for men and 22.3% for women.

### Increase of men

The process of the incorporation of men into the activity is very recent and has an unequal character in terms of the different areas of fishing and guilds of Galicia. In 2009, 90% of shellfishing on foot permits were granted to women. Ten years later, in 2019, this percentage was 70%. This progressive masculinization of the activity has been accompanied by a reduction in the number of permits by 12%, from 4281 in 2009 to 3777 in 2019 (Fig. 1).

Since 2009, the first year for which sex-disaggregated data are published, the number of licences held by men has grown by an average of 2.7 per cent per year. Their number has grown by 596 permits, from 9.2 of the total in 2009 to 30.2% in 2019. Although the figures reflect a change in the strong feminization of the activity, this situation is uneven across Galicia as there are significant differences at the local level. For statistical purposes, Galicia is divided into 9 fishing areas (Fig. 2), and the number of shellfishing on foot permits granted has fallen in all of them in the decade 2009–2019, with the exception of zone II, Pontevedra, where they have increased slightly.

**Fig. 3:**
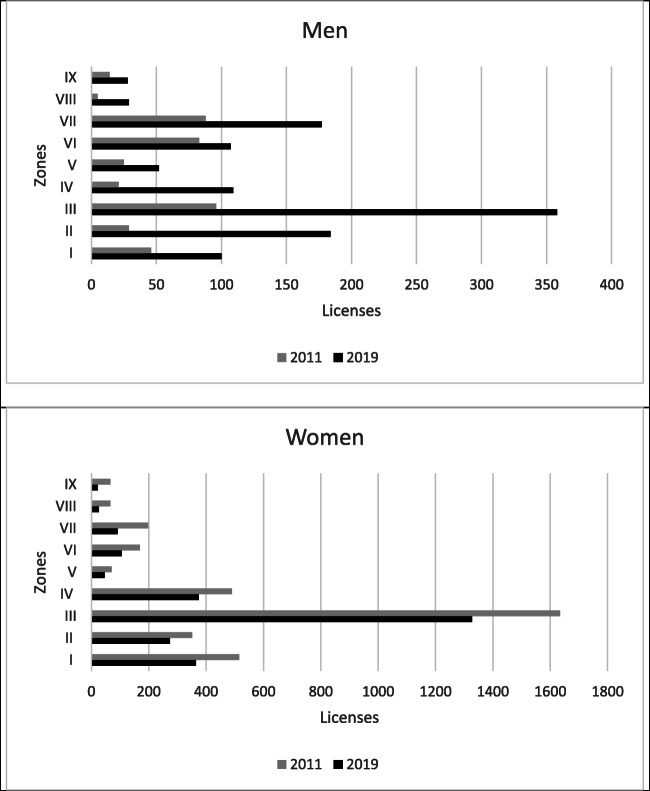
Permits for shellfish on foot by fishing areas in Galicia (2009–2019). Source: Galician Institute of Statistics. www.ige.eu

In this context, it is significant that in all areas, the number of permits granted to men increased and those granted to women decreased (Fig. 3). The highest volume of shellfishermen is concentrated in zone III Arousa with 358 licences, which, together with zones I and II (all three on the South Western coast of Galicia), account for 56% of permits granted to men for this activity in Galicia in 2011 and 69% of permits granted to men in 2019 (Table 1).

**Table 1:**
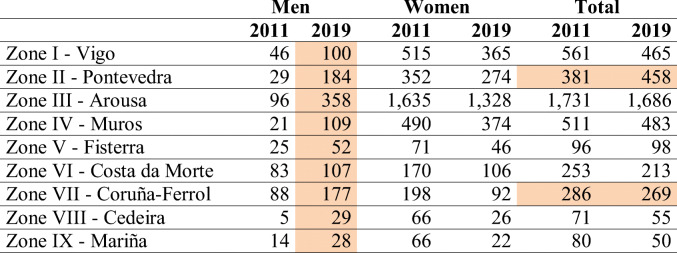
Evolution of shellfish walking permits by fishing areas in Galicia (2011–2019)

The areas with positive evolution are highlighted. Source: Galician Institute of Statistics. www.ige.eu

**Fig. 4.**
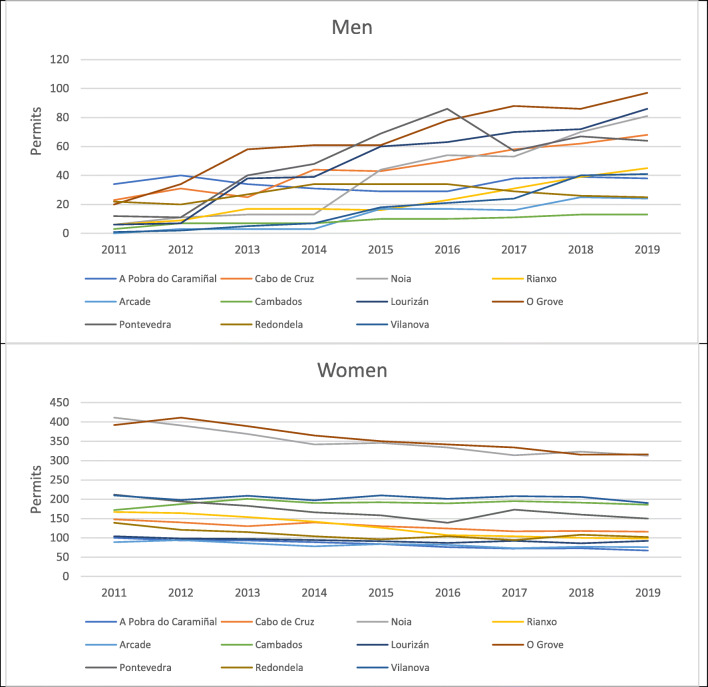
Annual evolution in guilds with more than 100 permits for shellfishing on foot in 2019 (2011–2019). Source: Galician Institute of Statistics. www.ige.eu

There are 62 fishermen’s associations in Galicia, 57 of which allow shellfish gathering on foot to be carried out in areas under their jurisdiction. However, there are 11 guilds that have more than 100 permits to carry out this activity; indeed, together they have 2521 permits, 66.7% of all in Galicia. A detailed analysis of the incorporation of men into these 11 guilds—in a context of a fall in the number of permits granted—allows us to see how the cost of this reduction has predominantly affected women. Even the rate at which women are leaving the sector exceeded the general rate of decline in the number of workers in the sector (Fig. 4).

### Changes in gendered valuation of the fisheries and fisheries labour

The conflicts that accompanied many shellfish areas in the second half of the twentieth century contributed to group cohesion and empowerment but also helped to create a problematic image of women shellfish workers. Martínez (2016) tells us about one of these conflicts, which took place at the end of Franco’s dictatorship, in the mid-1970s, on one of the most productive shellfish banks in Galicia. Attempts to privatize the area were met with strong and united opposition from the shellfish workers. Through semi-structured interviews, Martínez (2016) highlights how that resistance, accompanied by repression and delegitimization, manifested feelings of pride, cohesion, and collectiveness. In addition, the study shows that even before the regulation of the sector in the 1990s, there was self-management with unwritten rules to ensure the conservation and responsible management of resources (Oubiña Martínez 2018). In short, there was a consensus that revolved around group interests that reinforced their own identity as shellfish harvesters. In short, there was a consensus that revolved around group interests that reinforced their own identity as shellfish harvesters, because shellfish harvesting was a fundamental part of their domestic economies, since family care work made it impossible to migrate or to take up jobs far from home with very strict timetibles.

The consideration of shellfish gathering on foot as a “family aid”, and the fact that the activity was carried out by women, who also worked as housewives, contributed to men having little interest in this activity. Sequeiros (1995) analyses the reasons for the limited appreciation for this work. To the pejorative image of these professionals was added the low commercial value of their products and the reduced income obtained from their work. The average income obtained was estimated at 1200 euros per year, a situation aggravated by the lack of social protection and their scarce representation within the organizational structures of the sector, namely the fishermen’s associations (guilds). Although both the production of shellfishing on foot and the value of it have increased progressively in recent years, afloat shellfishing is an activity with much more lucrative returns, where there is an absolute preponderance of men. As a reference, in 2015, the average daily earnings for shellfish on foot was € 77, compared to the average daily earnings per boat in the same year, which was € 161 (MAPA; 2016).

The process of the professionalization of the sector and the economic crisis of the early 2000s has been key to the incorporation of men into this activity that has been traditionally associated with women. Most of the men that have joined the sector have come from professional sectors other than fishing, such as construction and other heavy industries, and have gained access through vacancies that have arisen with the retirement of older shellfisherwomen on foot (Gago 2016, *La Voz de Galicia*). They have obtained permits after a selection process that follows the completion of a specific training course for this modality of shellfishing, and in which priority is given to the local population, those who are unemployed, and those with higher levels of study and qualifications related to aquaculture production.

At present, the main challenges facing this process of the professionalization of shellfish farming on foot are, on the one hand, the need to increase workers’ incomes and, on the other, generational changes in a profession where the average age of workers is very high. Firstly, the incomes of people engaging in shellfishing on foot have increased considerably; in 1995 it was estimated to be increasing 1200 euros per year. Incomes vary substantially from one guild to another, depending on the wealth of the shellfish sandbanks and the value of the commercially available species. They range from almost 14,000 euros per year in the Cofradía of Cambados in 2016 to just over 12,000 euros in O Grove and Carril and 7200 euros in A Illa de Arousa (Estévez 2017, *La Voz de Galicia*). This income levels do not generally give workers complete economic independence (Frangoudes et al. 2008), a key aspect that will determine the continued incorporation of men into the activity.

Secondly, shellfishing on foot is an elderly profession. The figures reflect the decline in the number of permits granted since the beginning of the twentieth century, as well as retirements and terminations for various reasons, which have led to the development of an ageing workforce. Fifty-six per cent of women working in the sector are over 50, compared to 28 per cent of men in this age range. The incorporation of men is leading to the rejuvenation of the profession, as 35% of men with a shellfishing on foot permit are under 40, compared to 12% of women.

## Discussion and conclusions

In the heavily masculinized fishing sector in Galicia, women have been confined to those less lucrative, informal occupations that are seen as supporting the main extractive activity. These include repairing nets, shellfishing on foot, and selling catches, usually on a local scale. These less professionalized activities have been the subject of a process of planning since the last decade of the twentieth century, led by the regional administration with extensive planning and management powers. This process has been highly dependent on the arrival of European funds linked to the Common Fisheries Policy.

Shellfish extraction on foot has been one of the subsectors most affected by this new organization of activity. Its workers were traditionally engaged in the harvesting of molluscs in the intertidal strip, destined for self-consumption or barter, with a strong seasonal character and without any type of labour recognition or social protection. Today, following a process of professionalization that has taken place over several decades, they have become professionals engaged in activities of preparation, cultivation, maintenance, and surveillance on the shellfish banks, throughout the year. These workers have managed to see their labour rights recognized, their social benefits improve, and their salaries increase, although there is still a long way to go before shellfishing on foot can become the main source of income for a family. Some progress that remains to be seen are those related to the recognition of professional diseases and innovation aimed at improving uniforms, protective elements, and work tools.

As this activity has become more professional, men have begun to show an interest in it, which has resulted in a progressive increase in those seeking to obtain a shellfishing on foot permit. This widespread incorporation in Galicia is much more pronounced in some fishing areas and guilds and is determined by the level of income gained from shellfish gathering on foot and the work opportunities for shellfish gathering by boat and fishing. These are subsectors to which women are barred from joining, as reflected in the Occupational Survey (Ocupesca), which shows that women account for 5% of workers on boats operating in the national fishery and 0.4% in the European Union fisheries and are entirely absent from boats in international waters (Ocupesca, 2017).

The example we have dealt with in this text fits perfectly with other analyses in the academic literature. The lack of information and invisibility, the scarce social prestige, the nature of family support and the demands of domestic labour, and the subsequent need for the workplace to be near home are some of the factors that unite this feminized activity with many others, as was made clear in the theoretical framework. Furthermore, we have also seen how the professionalization of shellfish gathering on foot, due to the increase in the economic value of the species captured and the consequent need to establish regulations, has been empowering for women in this sector, while at the same time stimulating the incorporation of men who, following Bradley’s (1993) criteria, have moved beyond the infiltration stage to the invasion and, even in some cases, the takeover stage.

According to Lindsay (2007), we are not simply facing a process of substitution but a change in the status of the profession with obvious implications for gender relations. In this research, we have found that, although it is true that the path to professionalization served to empower women shellfish gatherers, it was also an instrument to exclude the most fragile from the system, i.e. those who were older, less educated, or with family responsibilities that were incompatible with undertaking “real” work. Many of these displaced women had been on the front line of the conflicts that arose as a result of the sector’s planning, such as those that were of a territorial nature, delineating the concession areas.

We consider it necessary to reflect on whether the regulation of shellfishing activity in the 1990s was actually a subtle form of control of women shellfish collectors and their work areas. Martínez (2016) points out that the discourse of the 1970s in favour of the privatization of shellfish sandbanks was justified as being less predatory and a more rational approach to the use of resources, exactly the same argument that accompanied the plans drawn up by the administrations 20 years later. In any event, as we have already seen, the results in human terms were, firstly, the exclusion of the most vulnerable women and, secondly, the progressive incorporation, infiltration, and invasion of men into the activity.

The empowerment of women shellfish harvesters from the 1990s onwards became stronger with their entry into the decision-making bodies of the guilds, with the recognition of their profession, and with the development of multiple initiatives ranging from tourism to the creation of their own organizations. Yet, the path was full of difficulties and was met with resistance Martínez (2019). Hence, Martínez (2016) states that professionalization has had an impact on this empowerment both at the individual and, to a lesser extent, the collective level. However, we believe that the empowerment of women shellfish workers is not the exclusive result of this process of the regulation of the activity but comes as the result of several different processes. In this sense, the fights started by the shellfish workers in the 1950s, and that intensified in the 1970s, to maintain the sandbanks as communal assets, in the face of privatization attempts, stand out as evidenced by the research of Martínez (2016).

While it is true that rural Galicia, at least since the middle of the nineteenth century, has been highly feminized due to emigration, with more than 80% of emigrants being male (Aldrey Vázquez 2006), in coastal areas, there were also absences, sometimes prolonged, of men because they were working on board ships. This gave women an unusual role in the economic sphere and in the occupation of public frontier spaces, such as sandbanks, that were usually reserved for men (Martínez, 2016). This anomaly, associated with organized and resistant women workers (Marugán Pintos 2004; Santasmarinas 2006), seems to be normalized with the professionalization of women shellfish harvesters and their consequent empowerment.

In this study, we have been able to verify that, although women’s empowerment is a necessary step to overcoming gender discrimination, it is far from sufficient to end the patriarchy. In fact, Martínez García (2016 and 2017) points out that once women’s participation in the power structures of the guilds is normalized, there are subtle and vaporous forms of control that are directly related to the systems of sex-gender domination. Furthermore, according to Scott (1990), there are also power relationships within the dominated groups where, once again, these gender inequalities are reproduced.

It is necessary, to evolve from the perspective of women in fisheries to gender in fisheries, in order to better understand the inequality in gender relations and how these are anchored to the structure of society and to provide the basis for more appropriate action (Williams et al. 2002; Williams 2008). Broullón Acuña (2011) interprets the process of the professionalization of women shellfish harvesters in terms of social control. The border territory, in this case the intertidal zones, is a space defined as conflictive and disorganized that administrations control in exchange for the participation of women shellfish gatherers in the exploitation of those spaces. According to Broullón Acuña (2011), this change also led to the evolution of this activity from occupying a subordinate role to a more central position, under the supervision of what this author calls expert agents. Although Broullón Acuña (2011) does not expressly cite the masculinization of the activity, there is a clear warning relating to how the patriarchal socio-sexual structure is controlling this process of regularization that is leading to the gradual displacement of women as a workforce. In this way, the self-appreciation of women’s empowerment without a strong gender awareness limits the potential for transformation (Martínez Vidal 2017).

In summary, at least two things have become evident from this study: firstly, that the work of shellfish catching fits perfectly with the socially established patterns of gender roles, by making it possible to combine professional and domestic work, and secondly, a process of empowerment has been described that, even with difficulties, has contributed to the normalization of the role of women in this activity. However, we have also tried to highlight the ways in which the patriarchy preserves the hegemonic position of men. In this sense, a progressive masculinization of this activity can be observed, without compensation in terms of the incorporation of women into the crews of fishing boats, which remained at 4.1% between 2011 and 2017 (Ocupesca, 2011, 2017), and in much more subtle ways that are protected by democratic ideas of justice in terms of access to shellfishing permits. Professionalization, associated with higher incomes and a greater time commitment on the part of workers, is leading directly to the substitution of the labour force and the recovery of public space, in this case the sandbanks, by men. Could it be that the empowerment of shellfishing women has generated a reaction based on a perceived attack on men and masculinity (Bannon and Correia 2006)? It is necessary to go deeper into this change, analysing in each one of the guilds the motivations that cause different degrees of incorporation of men into shellfishing on foot at a local level. It is also important to study in detail why women do not seem to follow the same path by engaging in shellfishing by boat, despite the fact that surveys suggest the availability of shipowners willing to hire them (CETMAR 2010). Finally, it is necessary to be aware that public policies must support greater equality in terms of employment opportunities in the fishing sector, and that, so far, there have been few initiatives in this regard.

Future research is required into the as yet under researched area regarding the incorporation of men into activities traditionally filled by women in the fishing sector. As has been said, the process of masculinization of shellfishing on foot is a relatively recent phenomenon, which has not been previously addressed in literature. The literature on the masculinization of feminized jobs has focused on professionalized fields, such as agriculture or nursing (Heggem 2014; Rochlen et al. 2009). In the case of shellfishing on foot, the process has been quite different since masculinization has occurred after professionalization.

The high economic value that many of the species that are caught are acquiring and the limited job opportunities in many small fishing villages point to a strengthening of the trend towards masculinization. If the 2008 crisis meant the transfer of many men who worked in construction to the hospitality industry and to other professions, such as shellfishing on foot, the COVID-19 crisis and its significant impact on the hospitality industry and tourism could accelerate the transfer of labour to sectors with increasing profitability, such as shellfishing on foot. These trends are the basis of future research that will try to delve into the implications of the masculinization of some fishing activities—such as shellfishing on foot—and of the stagnation of the incorporation of women into other strongly masculinized activities—such as fishing—for the understanding of gender roles in activities and communities highly dependent on fishing.

## Data Availability

Not applicable.
